# Community Awareness, Utilization, and Factors Influencing the Use of Over-the-Counter Scar Management Products: A Cross-Sectional Survey

**DOI:** 10.7759/cureus.117199

**Published:** 2026-09-30

**Authors:** Vidya Kiran Bammidi, Taskeen K Sethi, K Murali, Heena D Tiwari, Saurabh Shekhar, Shiprita Kumari, Anil Managutti

**Affiliations:** 1 Department of General Surgery, Gayatri Vidya Parishad Institute of Health Care and Medical Technology, Visakhapatnam, IND; 2 Department of Oral and Maxillofacial Surgery, Luxmi Bai Institute of Dental Sciences and Hospital, Patiala, IND; 3 Department of Burns, Plastic and Reconstructive Surgery, Kilpauk Medical College and Hospital, Chennai, IND; 4 Department of Blood Cell, Commissionerate of Health and Family Welfare, Government of Telangana, Hyderabad, IND; 5 Department of Oral Pathology and Microbiology, Faculty of Dentistry, Asian Institute of Medicine, Science and Technology (AIMST) University, Bedong, MYS; 6 Department of Anatomy, Faculty of Dentistry, Asian Institute of Medicine, Science and Technology (AIMST) University, Bedong, MYS; 7 Department of Oral and Maxillofacial Surgery, Narsinhbhai Patel Dental College and Hospital, Visnagar, IND

**Keywords:** community awareness, consumer behavior, over-the-counter products, scar management, self-medication, silicone gel

## Abstract

Introduction: Over-the-counter (OTC) scar management products are readily accessible and are frequently selected by individuals seeking improvements in the appearance or symptoms of scars. Community awareness, sources of information, perceptions of effectiveness and safety, and demographic characteristics may influence their utilization. This study assessed the awareness and utilization of OTC scar management products and identified factors associated with their use among adults.

Methods: This cross-sectional questionnaire-based survey was conducted among adults aged 18 years and above, who were recruited after obtaining informed consent. A structured questionnaire developed with multidisciplinary input from oral and maxillofacial surgeons, plastic surgeons, and general surgeons was used to assess the demographic characteristics, scar history, awareness, utilization patterns, sources of information, perceived outcomes, satisfaction, and factors influencing product selection. Categorical data are summarized as frequencies and percentages. Associations were evaluated using the chi-square or Fisher exact test, followed by binary logistic regression to identify independent predictors of OTC scar product use.

Results: Of the 700 participants, 600 completed the survey, yielding a response rate of 85.7%. A noticeable scar was reported by 451 (75.2%) participants, and 386 (64.3%) were aware of the availability of OTC scar management products. Among the participants with scars, 269 (59.6%) had previously used an OTC scar management product. Silicone gel was the most commonly recognized OTC product, reported by 201 (33.5%) participants, and was the most frequently used product among users. Social media was the leading initial source of information, reported by 179 participants (29.8%). Among the OTC product users, cosmetic improvement was the predominant reason for use, as reported by 108 (40.1%). Most users reported no adverse events. Improvement was reported as significant by 43 (16.0%), moderate by 98 (36.4%), and slight by 76 (28.3%). Multivariable logistic regression showed that higher education (adjusted odds ratio (AOR) = 2.18, 95% CI: 1.39-3.42; p = 0.001), urban residence (AOR = 1.74, 95% CI: 1.13-2.67; p = 0.012), awareness of OTC product availability (AOR = 5.63, 95% CI: 3.64-8.71; p < 0.001), belief in product effectiveness (AOR = 2.46, 95% CI: 1.62-3.73; p < 0.001), and receiving information from a doctor or pharmacist (AOR = 1.68, 95% CI: 1.05-2.67; p = 0.028) were independently associated with OTC scar product utilization.

Conclusion: OTC scar management products are widely recognized and commonly used among the surveyed adults, with silicone-based products being particularly prominent. Awareness, education, residence, perceived effectiveness, and professional sources of information were independently associated with utilization. Strengthening evidence-based community education and professional guidance may facilitate more appropriate and informed use of OTC scar management products.

## Introduction

Scarring is a natural consequence of wound healing following trauma, surgical procedures, burns, acne, infections, or other dermatological conditions [1]. Although scar formation is an essential part of tissue repair, many individuals experience cosmetic concerns, functional limitations, discomfort, itching, pain, and psychological distress related to visible scars [2]. The appearance of scars can negatively affect self-esteem, body image, and quality of life, particularly when scars involve exposed areas, such as the face and neck [3]. Consequently, effective scar management has become an important component of both medical treatment and patient-centered care.

A wide range of scar management modalities has been developed to improve scar appearance and minimize symptoms. These include silicone gels and sheets, onion extract-based formulations, vitamin E preparations, herbal products, pressure therapy, corticosteroids, laser therapy, and surgical scar revision [4,5]. Among these, over-the-counter (OTC) scar management products have gained considerable popularity because they are readily available without prescriptions, relatively affordable, convenient to use, and widely promoted through pharmacies, advertisements, social media, and online platforms [6]. Silicone-based products remain the most widely recommended first-line non-invasive treatment for the prevention and management of hypertrophic and immature scars, while numerous other topical products are marketed despite varying levels of clinical evidence supporting their effectiveness [4-6].

Despite the widespread availability of OTC scar management products, their appropriate selection and use may be influenced by individuals’ knowledge, attitudes, and perceptions of scar treatment. Patients increasingly encounter information and promotional claims regarding scar management products through online sources, where the advertised benefits may not always be supported by robust clinical evidence [7]. Consequently, professional guidance remains important to ensure appropriate product selection and realistic expectations regarding treatment outcomes [8]. Furthermore, factors such as age, education, socioeconomic status, accessibility, previous treatment experiences, and healthcare recommendations may significantly influence consumer behavior and treatment decisions. Understanding these determinants is essential for developing educational strategies that promote evidence-based scar management and encourage informed decision-making among the general public.

Although several studies have evaluated the clinical effectiveness of different scar management therapies, relatively few have investigated community-level awareness, utilization patterns, and factors influencing the use of OTC scar management products, particularly in developing countries where self-medication practices are common [4,9,10]. Information regarding public knowledge, sources, treatment preferences, perceived effectiveness, and satisfaction remains limited [9]. The aim of the present study was to assess community awareness and utilization of OTC scar management products and identify the factors influencing their use among adults. The objectives were to assess participants’ awareness and knowledge of available OTC scar management products and identify their major sources of information; evaluate patterns of product utilization, including the type of product used, reasons for use, duration of use, adherence, factors influencing product selection, self-reported adverse effects, perceived improvement, satisfaction, and willingness to recommend these products; and determine the demographic, awareness, and perception-related factors associated with OTC scar management product utilization among adults.

## Materials and methods

Study design and setting

A cross-sectional, questionnaire-based survey was conducted at Narsinhbhai Patel Dental College and Hospital, Visnagar, Gujarat, India, over a period of six months, from June to December 2022. The study was conducted after obtaining approval from the Institutional Ethics Committee (NPDCH/IEC/2022/082). All participants were informed of the purpose and nature of the study, and informed consent was obtained before administering the questionnaire. Participation was voluntary, and anonymity and confidentiality of the participants were maintained throughout the study.

Development of the questionnaire

A structured questionnaire was developed specifically for the present study after reviewing the available literature on scar management, self-medication, and OTC scar management products (see Appendix). To ensure that the questionnaire reflected perspectives from different specialties involved in the management of wounds and scars, its development involved clinicians working in General Surgery, Plastic and Reconstructive Surgery, and Oral and Maxillofacial Surgery at different institutions. The multidisciplinary composition of the expert group was intended to ensure that the questionnaire addressed general community behavior and clinically relevant aspects of scar management without disproportionately representing the perspective of a single specialty.

The questionnaire was designed in simple and easily understandable language, so that it could be administered to adults with different educational backgrounds. It consists predominantly of closed-ended questions and is divided into five sections (see Appendix, Table 7). The first section collected demographic information including age, sex, educational level, occupation, and place of residence. The second section assessed the participant's history and scar characteristics, including the presence of a noticeable scar, cause, anatomical location, and duration. The third section evaluated awareness and knowledge of OTC scar management products, including awareness of their availability, type of product known to the participant, source of information, perceived appropriate timing for initiation of scar treatment, perceived effectiveness, and perceived safety of using these products without professional consultation. The fourth section was directed toward participants who had previously used an OTC scar management product and assessed the type of product used, reasons for use, factors influencing purchase, duration of use, adherence to the manufacturer's instructions, adverse effects, and perceived improvement. The final section assessed overall satisfaction and willingness to recommend OTC scar management products to individuals with similar scars.

Questionnaire scoring and coding

As the principal outcomes of the study were awareness, utilization, behavioral patterns, and factors associated with OTC scar product use, the questionnaire was primarily analyzed using categorical response coding, rather than an overall knowledge or awareness score. Dichotomous responses such as “Yes” and “No” were coded as separate binary categories, while nominal and ordinal responses were assigned numerical codes for statistical entry and analysis. Awareness of OTC scar management products was principally determined from the participants’ response regarding prior awareness that scar management products could be obtained without a prescription. Utilization was defined as a positive response to the previous use of an OTC scar management product. Satisfaction and perceived improvement were retained as ordinal categories, ranging from very satisfied to very dissatisfied and from significant improvement to worsening of the scar, respectively. No arbitrary composite cut-off for “good” or “poor” knowledge was applied because the questionnaire was intended to characterize community awareness and patterns of use rather than function as a diagnostic knowledge scale.

Validation and pilot testing of the questionnaire

The initial questionnaire was subjected to expert assessment of content and face validity. Experts from General Surgery, Plastic and Reconstructive Surgery, and Oral and Maxillofacial Surgery (VKB, TKS, KM, and AM) reviewed individual questions for clinical relevance, appropriateness, clarity, comprehensiveness, and ability to adequately represent the domains of community awareness and OTC scar management practices. Suggestions provided by experts were incorporated into the questionnaire before the pilot administration.

A pilot study was subsequently conducted among 20 adults, representative of the intended study population, to assess the clarity, comprehensibility, wording, sequence, and ease of completion of the questionnaire. Participants involved in the pilot testing were asked to identify questions that were difficult to understand, ambiguous, repetitive, or potentially confusing. The necessary modifications were made based on the feedback obtained. Responses collected during the pilot testing were not included in the final statistical analysis. Internal consistency was assessed for the Likert-type items measuring participants’ perceptions of OTC scar management products. Cronbach’s α for this perception subscale was 0.84, indicating good internal consistency. Demographic variables and nominal categorical items, including product type and sources of information, were not included in the Cronbach’s α analysis.

Eligibility criteria

Adults aged ≥18 years who were willing to provide informed consent and complete the questionnaire were eligible for participation irrespective of the presence of a current or previous scar or prior awareness of OTC scar management products. Healthcare professionals directly involved in dermatology, plastic surgery, oral and maxillofacial surgery, or pharmacies were excluded to minimize the possibility that specialized professional knowledge would influence estimates of community-level awareness. Individuals who were unable to understand or complete the questionnaire and those with incomplete information were excluded from the final analysis. Duplicate responses were identified and excluded.

Sample size estimation

The sample size was estimated using G*Power software, version 3.1.9.7 (Heinrich Heine University Düsseldorf, Düsseldorf, Germany). As reliable prior estimates of community awareness regarding OTC scar management products were unavailable, an expected population proportion of 50% was selected, providing the most conservative estimate and maximum required sample size. At a 95% confidence level and margin of error of 5%, the minimum required sample size was calculated to be 385 participants. However, to improve the precision of the estimates, permit meaningful subgroup analyses according to demographic characteristics, and compensate for incomplete or non-returned questionnaires, the target sample size was increased to 700 participants.

Questionnaire administration and data collection

Eligible participants were approached using convenience sampling during the predefined three-month data-collection period from June to December 2022. Adults attending Narsinhbhai Patel Dental College and Hospital, including patients and accompanying attendants as well as eligible individuals from the surrounding community, were invited to participate. The potential participants were approached directly by trained investigators, provided with a brief explanation of the purpose and procedure of the study, and screened for eligibility. Those fulfilling the eligibility criteria and willing to participate were enrolled after they provided informed consent. The questionnaire was administered in a self-reported format, with assistance limited to the clarification of questions when required, and without influencing the participant's choice of response.

To facilitate a satisfactory response rate, the questionnaire was deliberately kept concise, predominantly close-ended, and written in a straightforward language. The participants were informed that their responses would remain anonymous and would be used exclusively for research purposes. Adequate time was provided for completion, and the completed questionnaires were checked for completeness at the time of collection whenever feasible. Participants who had not completed the questionnaire were reminded during the data collection period without exerting pressure to participate. These measures were intended to reduce the respondent burden, minimize missing information, and maximize the completeness of the returned questionnaires. After collection, the questionnaires were screened for eligibility, completeness, and duplicate entries before data entry.

Outcome measures

The primary outcome of the study was community awareness of the availability of OTC scar management products. Secondary outcomes included the prevalence and pattern of OTC scar product utilization, types of products used, sources of information, reasons for product use, factors influencing purchasing decisions, duration and adherence to product use, self-reported adverse effects, perceived improvement in scar appearance or symptoms, overall satisfaction, and willingness to recommend products to others. Demographic and awareness-related characteristics were additionally evaluated to determine the factors independently associated with OTC scar management product utilization.

Statistical analysis

Data obtained from the completed questionnaires were entered into Microsoft Excel (Microsoft Corporation, Redmond, WA, USA) and subsequently analyzed using IBM SPSS Statistics for Windows (version 25.0; IBM Corp., Armonk, NY, USA). Data were checked for completeness and consistency before the analysis. As the principal study variables were categorical, the descriptive findings were summarized using frequencies and percentages. Associations between demographic characteristics, awareness, knowledge-related variables, and utilization of OTC scar management products were initially evaluated using Pearson's chi-squared test. Fisher's exact test was applied when the expected cell frequencies were less than five. Variables demonstrating statistically significant associations during univariate analysis were subsequently entered into a binary logistic regression model to determine independent predictors of OTC scar management product use, while accounting for the simultaneous influence of other variables. The results of the multivariable analysis were expressed as adjusted odds ratios (AORs) with corresponding 95% confidence intervals (95% CIs). Model performance was assessed using the Nagelkerke R², Hosmer-Lemeshow goodness-of-fit test, and overall classification accuracy. Before fitting the multivariable logistic regression model, multicollinearity among the candidate predictors was assessed using variance inflation factor (VIF) and tolerance values; no problematic multicollinearity was identified. All statistical tests were two-tailed, and statistical significance was set at p < 0.05.

## Results

A total of 700 participants were approached for the study, of whom 600 completed the questionnaire, resulting in a response rate of 85.7%. Among the respondents, 314 (52.3%) were females, and 286 (47.7%) were males. The largest proportion belonged to the 18-30-year age group, followed by the 31- 45-year age group. More than half of the participants had higher education, while 221 (36.8%) had basic education. Regarding occupation, 243 (40.5%) participants were working, and 176 (29.3%) were students. Most participants were urban residents (Table 1).

**Table 1:** Demographic characteristics of the study participants (N = 600). Data are presented as n (%). N: total number of participants.

Variable	Category	n (%)
Age group	18–30 years	236 (39.3)
	31–45 years	184 (30.7)
	46–60 years	122 (20.3)
	>60 years	58 (9.7)
Sex	Male	286 (47.7)
	Female	314 (52.3)
Education	No formal education	49 (8.2)
	Basic education	221 (36.8)
	Higher education	330 (55.0)
Occupation	Student	176 (29.3)
	Working	243 (40.5)
	Homemaker	121 (20.2)
	Retired	60 (10.0)
Residence	Urban	366 (61.0)
	Rural	234 (39.0)

Of the 600 participants, 451 (75.2%) reported a history of scarring. Among these participants, accidents or trauma were the most common cause, followed by surgical procedures and acne. The face was the most frequently affected site, followed by the lower and upper limbs. Scars were present for more than one year in 226 (50.1%) participants. Overall, 386 (64.3%) respondents were aware that scar management products were available over the counter, while 214 (35.7%) were unaware of their availability (Table 2).

**Table 2:** Scar characteristics and awareness of over-the-counter scar management products (N = 600). Data are presented as n (%), Percentages for scar cause, location, and duration are based on participants with a noticeable scar (n = 451). N: total number of participants, OTC: over-the-counter

Variable	Category	n (%)
History of noticeable scar	Yes	451 (75.2)
	No	149 (24.8)
Primary cause of scar (n = 451)	Surgical procedure	95 (21.1)
	Accident/trauma	170 (37.7)
	Burn	45 (10.0)
	Acne	88 (19.5)
	Infection	31 (6.9)
	Other	22 (4.9)
Scar location (n = 451)	Face	126 (27.9)
	Neck	18 (4.0)
	Upper limb	94 (20.8)
	Lower limb	103 (22.8)
	Chest/Abdomen	49 (10.9)
	Back	34 (7.5)
	Other	27 (6.0)
Scar duration (n = 451)	<3 months	44 (9.8)
	3–6 months	71 (15.7)
	6–12 months	110 (24.4)
	>1 year	226 (50.1)
Aware OTC products are available	Yes	386 (64.3)
	No	214 (35.7)

Regarding knowledge and awareness of OTC scar management products, silicone gel was the most commonly recognized product, followed by scar cream and onion extract gel. Social media was the most frequently reported first source of information, followed by family or friends, pharmacists, and Internet searches. Regarding the timing of scar management, 254 (42.3%) participants believed that treatment should be initiated immediately after wound closure, whereas 206 (34.3%) considered treatment appropriate after complete healing. Approximately half of the respondents believed that the OTC scar management products were effective. However, only 241 (40.2%) participants considered these products safe for use without consultation with a healthcare professional (Table 3).

**Table 3:** Knowledge and awareness regarding over-the-counter scar management products (N = 600). Data are presented as n (%). N: total number of participants, OTC: over-the-counter

Variable	Category	n (%)
Best known OTC product	Silicone gel	201 (33.5)
	Silicone sheets	61 (10.2)
	Scar creams	165 (27.5)
	Onion extract gel	76 (12.7)
	Herbal products	44 (7.3)
	Vitamin E	18 (3.0)
	Not aware	35 (5.8)
First source of information	Doctor/Dentist	78 (13.0)
	Pharmacist	91 (15.2)
	Family/Friends	96 (16.0)
	Social media	179 (29.8)
	Television/Newspaper	49 (8.2)
	Internet search	89 (14.8)
	Other	18 (3.0)
Ideal time to start treatment	Immediately after wound closure	254 (42.3)
	After complete healing	206 (34.3)
	When scar becomes visible	78 (13.0)
	Don't know	62 (10.3)
Believe OTC products are effective	Yes	311 (51.8)
	No	289 (48.2)
Believe OTC products are safe without consultation	Yes	241 (40.2)
	No	359 (59.8)

Among 451 participants with a history of scars, 269 (59.6%) reported having used an OTC scar management product, whereas 182 (40.4%) had never used one. Among users, silicone gel was the most frequently used product, followed by scar cream and onion extract gel. Improvement in cosmetic appearance was the most frequently reported reason for product use, followed by reduction in itching and a doctor's recommendation. Doctor recommendation was also the most influential factor in product-purchasing decisions, followed by pharmacist recommendation, brand reputation, and price. Most users reported using the product for one to three months, followed by three to six months (Table 4).

**Table 4:** Patterns of OTC scar management product utilization among participants with scars (n = 451), with product-specific items reported among users (n = 269). Data are presented as n (%). Product type, reason for use, purchasing factor, and duration of use are reported among participants who had used an OTC scar product (n = 269). OTC: over-the-counter

Variable	Category	n (%)
Ever used OTC scar product	Yes	269 (59.6)
	No	182 (40.4)
Type of product used (n = 269)	Silicone gel	112 (41.6)
	Silicone sheet	28 (10.4)
	Scar cream	70 (26.0)
	Onion extract gel	33 (12.3)
	Herbal product	18 (6.7)
	Vitamin E	8 (3.0)
Reason for use	Cosmetic appearance	108 (40.1)
	Reduce itching	46 (17.1)
	Reduce pain	21 (7.8)
	Doctor recommendation	42 (15.6)
	Family/Friend recommendation	25 (9.3)
	Prevent scar formation	27 (10.0)
Most influential purchasing factor	Doctor recommendation	59 (21.9)
	Pharmacist recommendation	48 (17.8)
	Brand reputation	42 (15.6)
	Price	39 (14.5)
	Advertisement	28 (10.4)
	Online reviews	33 (12.3)
	Family/Friends	20 (7.4)
Duration of use	<1 month	43 (16.0)
	1–3 months	116 (43.1)
	3–6 months	72 (26.8)
	>6 months	38 (14.1)

Among the OTC scar product users, 164 (61.0%) reported always following the manufacturer's instructions, whereas 78 (29.0%) followed them sometimes. Most users reported no adverse events. Among the reported adverse effects, skin irritation and redness were the most frequent. Regarding perceived treatment outcomes, 98 (36.4%) participants reported moderate improvement, 76 (28.3%) reported slight improvement, 43 (16.0%) reported significant improvement, and 45 (16.7%) reported no improvement. Overall, 109 (40.5%) users were satisfied, and 41 (15.2%) were very satisfied with the products. Furthermore, 111 (41.3%) stated that they would probably recommend OTC scar management products to others with similar scars, whereas 65 (24.2%) would definitely recommend them (Table 5).

**Table 5:** Treatment outcomes and user perceptions among over-the-counter scar product users (n = 269). Data are presented as n (%). OTC: over-the-counter

Variable	Category	n (%)
Used according to instructions	Always	164 (61.0)
	Sometimes	78 (29.0)
	Never	27 (10.0)
Adverse effects	None	219 (81.4)
	Skin irritation	17 (6.3)
	Redness	15 (5.6)
	Allergy/Rash	12 (4.5)
	Pigmentation	4 (1.5)
	Other	2 (0.7)
Improvement noticed	Significant	43 (16.0)
	Moderate	98 (36.4)
	Slight	76 (28.3)
	No improvement	45 (16.7)
	Worse	7 (2.6)
Overall satisfaction	Very satisfied	41 (15.2)
	Satisfied	109 (40.5)
	Neutral	67 (24.9)
	Dissatisfied	37 (13.8)
	Very dissatisfied	15 (5.6)
Recommend to others	Definitely yes	65 (24.2)
	Probably yes	111 (41.3)
	Not sure	46 (17.1)
	Probably not	35 (13.0)
	Definitely not	12 (4.5)

Univariate analysis demonstrated significant associations between OTC scar management product use and higher education (p < 0.001), urban residence (p = 0.001), awareness of OTC product availability (p < 0.001), belief in product effectiveness (p < 0.001), and receiving information from doctors or pharmacists (p = 0.001). In multivariable binary logistic regression analysis, awareness of OTC product availability emerged as the strongest independent predictor of utilization (adjusted odds ratio (AOR) = 5.63, 95% confidence interval (CI: 3.64-8.71; p < 0.001). Participants who believed that OTC scar products were effective were also more likely to use them (AOR = 2.46, 95% CI: 1.62-3.73; p < 0.001). Higher education was independently associated with greater utilization (AOR = 2.18, 95% CI: 1.39-3.42; p = 0.001), as was urban residence (AOR = 1.74, 95% CI: 1.13-2.67; p = 0.012). Participants who received information from a doctor or pharmacist also had significantly greater odds of OTC product use (AOR = 1.68, 95% CI: 1.05-2.67; p = 0.028). The final regression model demonstrated a Nagelkerke R² of 0.41, a non-significant Hosmer-Lemeshow goodness-of-fit test (p = 0.71), and an overall classification accuracy of 78.4% (Table 6).

**Table 6:** Factors associated with over-the-counter scar management product use among participants with scars (n = 451). Associations in the univariate analysis were assessed using the chi-square test; independent predictors were evaluated using multivariable binary logistic regression; *p < 0.05 was considered statistically significant. No higher education (includes basic education and no formal education). Other sources (includes family/friends, social media, television/newspaper, Internet search, and other). Model fit: Nagelkerke R² = 0.41, Hosmer–Lemeshow test, p = 0.71; classification accuracy = 78.4%. AOR: adjusted odds ratio, CI: confidence interval, OTC: over-the-counter

Variable	Category	Used OTC n (%)	Did not use n (%)	Univariate analysis (Chi-square test)	p-value	Multivariate logistic analysis	95% CI	p-value
		(n = 269)	(n = 182)	χ² statistic		Adjusted OR		
Education	Higher education	178 (66.2)	76 (41.8)	25.84	< 0.001*	2.18	1.39–3.42	0.001*
	No higher education (reference)	91 (33.8)	106 (58.2)			1 (Reference)		
Residence	Urban residence	183 (68.0)	96 (52.7)	10.92	0.001*	1.74	1.13–2.67	0.012*
	Rural (reference)	86 (32.0)	86 (47.3)			1 (Reference)		
Awareness of OTC products	Aware OTC product available	231 (85.9)	89 (48.9)	70.61	< 0.001*	5.63	3.64–8.71	< 0.001*
	Not aware (reference)	38 (14.1)	93 (51.1)			1 (Reference)		
Belief in product effectiveness	Believe products are effective	181 (67.3)	68 (37.4)	39.87	< 0.001*	2.46	1.62–3.73	< 0.001*
	Do not believe (reference)	88 (32.7)	114 (62.6)			1 (Reference)		
Information source	Doctor/Pharmacist	103 (38.3)	42 (23.1)	11.84	0.001*	1.68	1.05–2.67	0.028*
	Other sources (reference)	166 (61.7)	140 (76.9)			1 (Reference)		

## Discussion

This study explored community awareness, utilization, perceptions, and factors influencing the use of OTC scar management products. Overall, awareness of these products was relatively high, and their use was common among individuals with a history of scars. Nevertheless, the findings also revealed important gaps between awareness, perceived effectiveness, safety perceptions, and actual utilization. This distinction is important because the availability of scar products without prescription may encourage self-directed treatment, whereas appropriate scar management depends on scar type, stage of healing, product selection, timing of intervention, and adherence.

Silicone gel has emerged as the most recognized and frequently used scar management product. This finding is consistent with previous literature supporting silicone-based therapy as one of the principal noninvasive approaches for the prevention and management of abnormal scars [5]. Silicone gels and sheets have been incorporated into international scar management recommendations, particularly for hypertrophic scars and keloids [10,11]. Their popularity may be explained by ease of application, availability without a prescription, relatively favorable safety profile, and greater professional familiarity compared to many alternative topical preparations. In contrast, products containing vitamin E, herbal ingredients, and other marketed scar-reducing compounds have variable levels of supporting evidence [6]. Therefore, the observed preference for silicone-based products may represent a combination of consumer familiarity, commercial availability, and healthcare professional recommendations.

Social media was the predominant source through which participants became aware of OTC scar management products. This reflects the increasing role of digital media in shaping health-related knowledge and consumer behavior [12]. Cosmetic and dermatological products are extensively promoted through social networking platforms, online advertisements, influencers, and consumer testimonials, making these sources readily accessible to the general population [12,13]. However, information obtained through social media may vary considerably in terms of scientific accuracy and may create unrealistic expectations regarding the extent or speed of scar improvement. Interestingly, recommendations from doctors and pharmacists remain important determinants of purchasing behavior. This suggests that, although digital platforms may facilitate initial product awareness, professional advice continues to play an important role in actual treatment decisions [14].

Cosmetic improvement was the predominant motivation for using the OTC scar products. This is understandable because visible scars, particularly those involving exposed anatomical regions, can affect body image, self-confidence, social interaction, and the overall quality of life. Previous studies have demonstrated that the burden of scarring extends beyond physical symptoms and may have considerable psychosocial consequences [3]. In addition, itching and discomfort have been reported as reasons for treatment, highlighting that individuals may seek OTC products for both esthetic and symptomatic improvement [15]. The predominance of traumatic and surgical scars in the study population may explain the considerable interest in readily accessible scar management strategies.

Another noteworthy finding was the generally favorable experience among individuals who used OTC products. Most users perceived some degree of improvement, and their overall satisfaction and willingness to recommend these products were relatively favorable. Most participants reported no adverse effects. These findings are broadly consistent with evidence indicating that commonly used topical scar-management interventions, particularly silicone-based preparations, are generally well tolerated and may improve several scar characteristics [5,16]. However, perceived improvement should be interpreted cautiously because scar appearance can change naturally during remodeling and maturation processes. Without an objective scar assessment or an untreated comparison group, improvement cannot be attributed exclusively to the product used.

Awareness of the availability of OTC products was strongly associated with product utilization in the multivariable analysis. Higher educational attainment, urban residence, belief in product effectiveness, and receipt of information from healthcare professionals were also independently associated with use. Greater utilization among highly educated participants may reflect better access to health information, greater health literacy, and increased familiarity with self-care products. Similarly, urban residents might have easier access to pharmacies, dermatological services, retail outlets, and online health information. The association between perceived effectiveness and utilization is also expected, as individuals who believe that a treatment is beneficial are more likely to initiate and continue its use [17]. Importantly, the association with professional sources of information emphasizes the continuing influence of clinicians and pharmacists in guiding consumer decisions [18].

Clinical implications

These findings highlight gaps in community knowledge regarding OTC scar management and underscore the importance of evidence-based information and appropriate professional guidance regarding product selection, timing of initiation, duration of application, realistic treatment expectations, and situations requiring specialist evaluation. Pharmacists may also contribute to informed decision-making when OTC products are obtained without prior clinical consultation. Reliable digital health information may provide an additional source of evidence-based information regarding scar management. These implications should be interpreted as potential areas for improving informed decision-making rather than evidence that educational or professional interventions would directly improve treatment outcomes, as such interventions were not evaluated in this cross-sectional study.

Limitations and recommendations

This study has several limitations. The cross-sectional design precludes the establishment of temporal or causal relationships between awareness-related factors and product utilization. In particular, both prior awareness (Q5) and previous product use (Q11) were assessed retrospectively by self-report; therefore, participants may have over-reported or inconsistently recalled whether awareness preceded their initial product use, introducing the possibility of recall and consistency bias. Consequently, the observed association between prior awareness and utilization should not be interpreted as evidence that awareness temporally preceded or caused product use. Information regarding product use, adverse effects, improvement, and satisfaction was self-reported and, therefore, susceptible to recall and response bias. Scar severity and treatment outcomes were not objectively assessed using validated clinical scar scales, and the different scar types were analyzed collectively. The findings were obtained from a defined study population and may, therefore, have limited generalizability to populations with different demographic or healthcare-access characteristics. Future multicenter studies involving geographically and socioeconomically diverse populations are warranted. Prospective studies incorporating validated scar assessment instruments and objective clinical evaluations would also help determine whether the perceived benefits of OTC products correspond to measurable improvements in scar characteristics.

## Conclusions

Community awareness and utilization of OTC scar management products are substantial, with silicone-based preparations being particularly prominent. Social media plays an important role in creating awareness, whereas healthcare professionals remain influential in product selection and utilization. Education, residence, awareness, and perception of effectiveness are important determinants of use. These findings highlight the need for accessible, evidence-based community education and greater professional guidance to promote the appropriate, safe, and realistic use of OTC scar management products.

## Appendices

**Table 7:** Study questionnaire.

Part 1- Demographic information			
1		Age group	□18-30, □ 31-45, □ 46-60, □ > 60
2		Sex	□ Male. □ Female
3		Education	□ No formal education, □ Basic, □ Higher
4		Occupation	□ Student, □ Working, □ Home maker, □ Retired
5		Residence	□ Urban, □ Rural
Part 2- Information about scar			
1	Have you ever had a noticeable scar?		□ Yes □ No
2	What was the primary cause of your scar?		□ Surgical procedure □ Accident/trauma □ Burn □ Acne □ Infection □ Other
3	Where is/was the scar located?		□ Face □ Neck □ Upper limb □ Lower limb □ Chest/abdomen □ Back □ Other
4	How long have you had the scar?		□ Less than 3 months □ 3–6 months □ 6–12 months □ More than 1 year
Part 3- Awareness of OTC scar management			
5	Before today, were you aware that scar management products can be purchased without a prescription?		□ Yes □ No
6	Which OTC scar management products are you aware of? (Select one that you know best)		□ Silicone gel □ Silicone sheets □ Scar creams □ Herbal preparations □ Onion extract gel □ Vitamin E products □ I am not aware of any
7	Where did you first hear about OTC scar management products?		□ Doctor/Dentist □ Pharmacist □ Family/Friends □ Social media □ Television/Newspaper □ Internet search □ Other
8	In your opinion, when should scar treatment ideally be started?		□ Immediately after wound closure □ After complete healing □ Only if scar becomes visible □ Don't know
9	Do you believe OTC scar management products are effective in improving scars?		□ Yes □ No
10	Do you think OTC scar products are safe to use without consulting a healthcare professional?		□ Yes □ No
Part 4- OTC medication use			
11	Have you ever used an OTC scar management product?		□ Yes □ No
12	Which type of OTC scar management product did you use?		□ Silicone gel □ Silicone sheet □ Scar cream □ Herbal product □ Onion extract gel □ Vitamin E product □ Other
13	What was the main reason for using the product?		□ Improve cosmetic appearance □ Reduce itching □ Reduce pain □ Doctor recommended □ Family/Friend recommendation □ Prevent scar formation
14	Which factor most influenced your purchase?		□ Doctor recommendation □ Pharmacist recommendation □ Brand reputation □ Price □ Advertisement □ Online reviews □ Recommendation from family/friends
15	For how long did you use the product?		□ Less than 1 month □ 1–3 months □ 3–6 months □ More than 6 months
16	Did you use the product according to the manufacturer's instructions?		□ Always □ Sometimes □ No
17	Did you experience any adverse effects?		□ No □ Skin irritation □ Redness □ Allergy/rash □ Increased pigmentation □ Other
18	Did you notice improvement in your scar?		□ Significant improvement □ Moderate improvement □ Slight improvement □ No improvement □ Scar worsened
Part 5- Perception and future use			
19	Overall, how satisfied were you with the OTC scar management product?		□ Very satisfied □ Satisfied □ Neutral □ Dissatisfied □ Very dissatisfied
20	Would you recommend OTC scar management products to others with a similar scar?		□ Definitely yes □ Probably yes □ Not sure □ Probably not □ Definitely not
